# Ipsilateral partial anomalous pulmonary venous connection in right lung cancer with absent right upper lobe

**DOI:** 10.1186/s12957-015-0524-7

**Published:** 2015-03-13

**Authors:** Zhengcheng Liu, Rusong Yang, Feng Shao, Yanqing Pan

**Affiliations:** Department of Thoracic Surgery, Nanjing Chest Hospital Affiliated to Southeast University, Nanjing, 210029 China

**Keywords:** PAPVC, Lung cancer, Pneumonectomy

## Abstract

A partial anomalous pulmonary venous connection (PAPVC) is an uncommon congenital anomaly. This report documents the case of a 48-year-old man with PAPVC which was incidentally discovered with right lung cancer and absence of right upper lobe. Right pneumonectomy was successfully performed, and the patient had an uneventful postoperative course. Asymptomatic PAPVC without septal defect is extremely rare. If the PAPVC is located in a different lobe, a pulmonary resection for lung cancer would precipitate an adverse outcome without a correction of the PAPVC. Surgeons should therefore be cautious regarding the potential existence of a PAPVC when a patient undergoes surgical procedures.

## Background

Surgical treatment should be selected for primary non-small cell lung cancer. However, congenital anomalous conditions are sometimes found during an operation. Partial anomalous pulmonary vein connection (PAPVC) is characterized by drainage of one or more pulmonary veins into the right atrium or one of its tributary veins. Major lung resection may cause acute right heart failure with large left-to-right shunt. We report a case of ipsilateral PAPVC with right lung cancer and absent right upper lobe managed by pneumonectomy. Research carried out was approved by ethics committee of Nanjing Chest Hospital in compliance with the Helsinki Declaration.

## Case presentation

**Figure 1:**
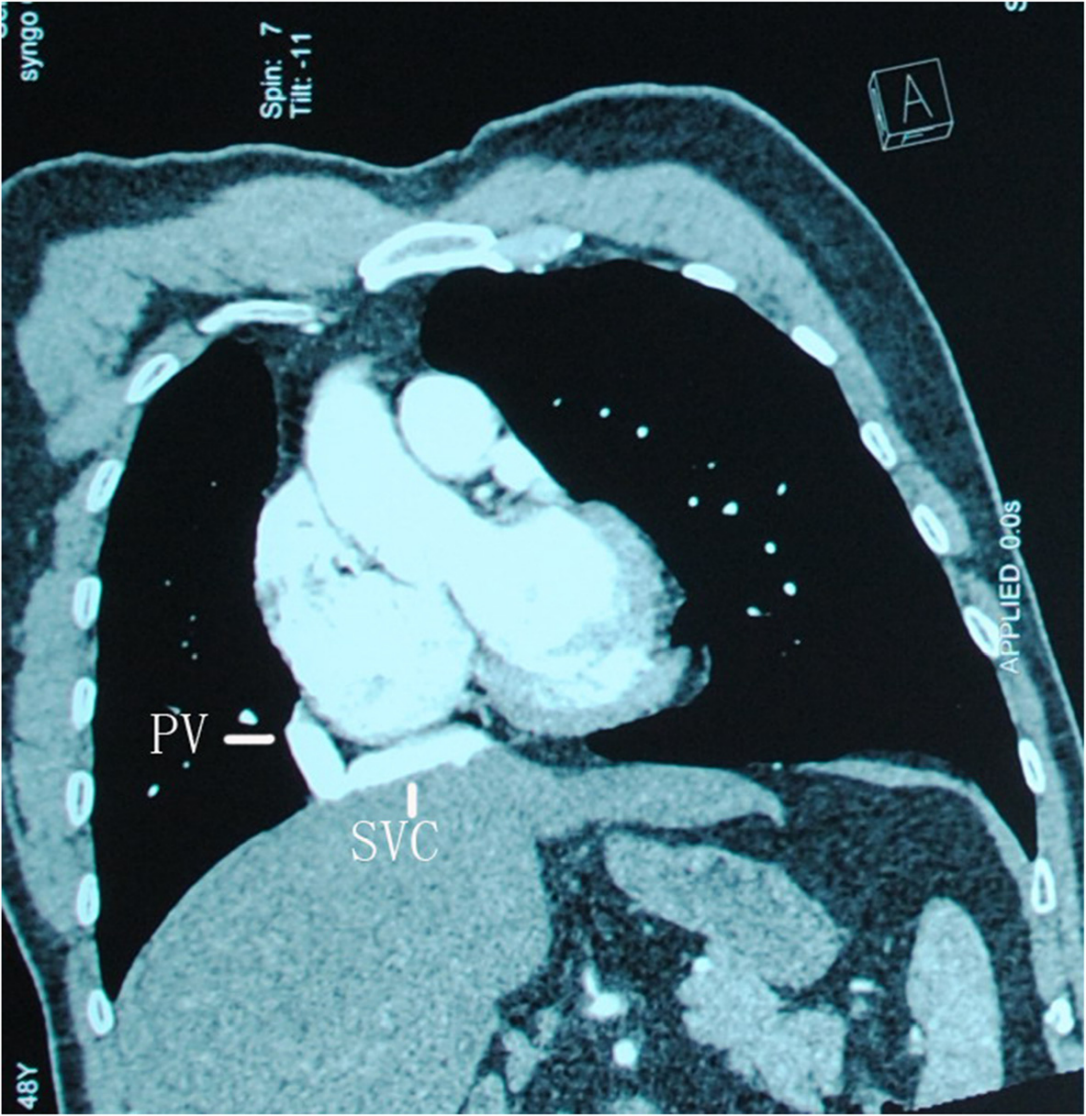
**Contrast-enhanced CT showing the right pulmonary vein (PV) draining into the inferior vena cava (IVC).**

A 48-year-old man was admitted to our institute with worsening cough and blood sputum. Enhanced chest computed tomography revealed a hilar mass in the right lower lobe with no mediastinal lymph node swelling and only one pulmonary vein on the right hilar site draining into the inferior vena cava, right superior bronchus, and pulmonary artery was absent (Figure 1). The abnormality as a PAPVC was diagnosed. Fiberoptic bronchoscopy revealed occlusion of the B6-10 bronchus by an endobronchial lesion with absence of B1-3 bronchus. Transbronchial biopsy revealed squamous cell carcinoma.

Echocardiogram showed normal left ventricular function with 65% ejection fraction; the right ventricle systolic pressure was 35 mmHg; and no remarkable abnormality such as an atrial septal defect was present. A pulmonary function study showed a forced expiratory volume in 1 s of 75% of the predicted value, a forced vital capacity of 96% of the predicted value, and a carbon monoxide diffusing capacity of 60% of the predicted value. An arterial blood gas analysis revealed a PaO_2_ of 87 mmHg and a PaCO_2_ of 45 mmHg in room air.

**Figure 2:**
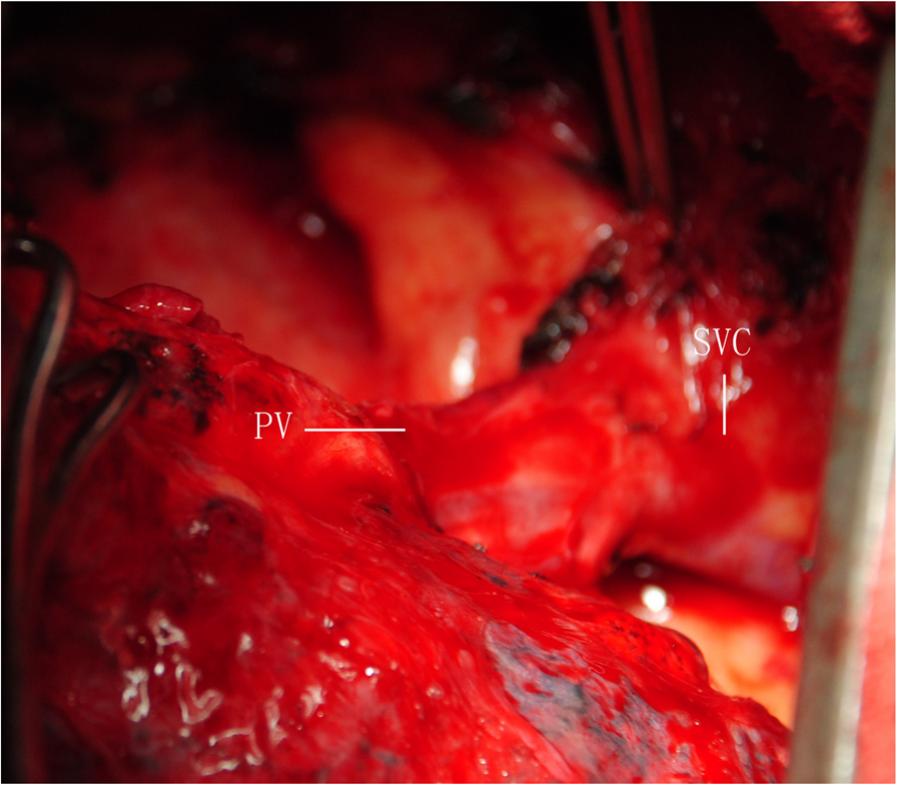
**The anomalous pulmonary vein (PV) was found draining into inferior vena cava (IVC).**

The planned operation for his lung cancer was a right pneumonectomy due to absence of right upper lobe and the abnormal pulmonary vein connection. The anomalous pulmonary vein was found draining into inferior vena cava; it was divided at its insertion point (Figure 2). His postoperative course was uneventful without any cardiac or respiratory failure. Pathological stage was IIIA (T2a N2 M0) for lung cancer. An arterial blood gas analysis revealed a PaO_2_ of 83 mmHg and a PaCO_2_ of 40 mmHg in room air.

The patient received radiation therapy and four cycles of chemotherapy; the patient was in good health with good exercise tolerance 2 years after operation. Ejection fraction was 63% measured by echocardiogram; the right ventricle systolic pressure was 38 mmHg.

PAPVC includes those cardiovascular anomalies in which one to three pulmonary veins connect to the right atrium directly or indirectly by way of different systemic venous connections. It occurs in about 0.6% to 0.7% of the population [1], according to autopsy data, but the actual incidence could be greater [2]. PAPVC can occur as an isolated anomaly, although it is commonly associated with a sinus venosus type of atrial septal defect (ASD). Some reports have suggested that PAPVC occurs approximately 10 times more frequently in the right pulmonary vein than in the left pulmonary vein [3]. Most cases of right PAPVC are likely to connect to the superior vena cava or right atrium. In this case, it connected to the inferior vena cava.

PAPVC without an atrial septal defect is possibly asymptomatic and clinically insignificant. When the PAPVC is located in the resected lobe, no hemodynamic problems should occur during the procedure. In this case, based on patient’s cardiac considerations and the ipsilateral lung cancer and PAPVC with absence of right upper lobe, pneumonectomy was performed without surgical correction of vessel anomaly. However, when the anomalous vein is located in the other lobe, serious complications may occur, such as right ventricular heart failure caused by increased left-to-right shunt flow [4]. Black and associates reported a patient with fatal right heart failure after right pneumonectomy for lung cancer with a missed contralateral PAPVC [5]. Besides, the surgical treatment of PAPVC has been recommended for patients with a Qp/Qs greater than 2.0, regardless of associated cardiac defects [6]. Generally, a PAPVC on the left side is corrected by a simple end-to-side anastomosis to the left auricular appendage or the left atrium or end-to-end anastomosis to the stump of the resected normal pulmonary vein without extracorporeal circulation [7]. When the PAPVC is located on the right side, cardiopulmonary bypass is usually required due to the shortness of the anomalous vein. Sakurai and associates reported a case of right PAPVC repair using total cardiopulmonary bypass before left pneumonectomy for lung cancer [8]. The treatment strategy depends on the location of the PAPVC.

Therefore, the preoperative discovery of asymptomatic PAPVC would be very important for patients with planned lung resection. Careful interpretation of the findings of the existing architectural structure is needed, including pulmonary artery, vein, or bronchus, as well as a tumor on the chest computed tomographic scan. Most of the findings of PAPVC can be identified by enhanced chest computed tomography. Conversely, if the findings of right heart overload are preoperatively detected, heart defects such as atrial septal defect or PAPVC should be considered. If patients with an asymptomatic PAPVC require major lung resection, the PAPVC should be corrected before lung resection to prevent fatal postoperative heart failure.

## Conclusions

We have described a rare case of an ipsilateral PAPVC with absence of right upper lobe and lung cancer. This report highlights the fact that although PAPVC in an adult is uncommon, it should be appropriately managed during pulmonary resection.

## Consent

Written informed consent was obtained from the patient for publication of this case report and any accompanying images. A copy of the written consent is available for review by the Editor-in-Chief of this journal.

## Abbreviation

PAPVC: partial anomalous pulmonary venous connection
